# Extra-lobar Pulmonary Sequestration Requiring Intrauterine Thoracentesis

**Published:** 2015-01-01

**Authors:** Ufuk Cakir, Dilek Kahvecioglu, Serdar Alan, Duran Yildiz, Hasan Akduman, Omer Erdeve, Gulnur Gollu Bahadir, Meltem Kologlu, Begum Atasay, Saadet Arsan

**Affiliations:** 1 Division of Neonatology, Department of Pediatrics, Ankara University School of Medicine, Ankara, Turkey.; 2 Neonatal Intensive Care Unit, Hitit University Corum Training and Research Hospital, Corum, Turkey.; 3 Department of Pediatric Surgery, Ankara University School of Medicine, Ankara, Turkey.

**Keywords:** Extra-lobar pulmonary sequestration, Fetal pleural effusion, Congenital lung malformations

## Abstract

Congenital lung malformations can result in significant morbidity and mortality in children. Pulmonary sequestration is an uncommon congenital malformation of the lung that can cause complications even in fetal life. We herein present a newborn with extra-lobar sequestration (ELS) that lead to hydrops fetalis necessitating fetal intervention.

## CASE REPORT

A fetus of 30 - week gestation developed pleural effusion which was detected on antenatal ultrasound (US). Fetal pleural effusion was drained consecutively on 30th, 31st and 32nd weeks of gestation under ultrasound guidance. Biochemical analysis of effusion sample was consistent with transudate. Congenital TORCH infections were ruled out by using polymerase chain reaction (PCR). Fetal anemia was ruled out by demonstration of normal middle cerebral artery-peak systolic volume (MCA-PSV) and congenital heart defects were excluded by fetal echocardiography. At 34 - week gestation, a male infant (2745 g) was born to a 24-year old mother by cesarean section, with APGAR scores of 6 and 7, at 1st and 5th minutes, respectively. The patient did not require any resuscitation in the delivery room. Later on, he was admitted to the neonatal intensive care unit (NICU) due to respiratory distress. Tachypnea, intercostal retractions, and decreased breath sounds on the left side of the chest were detected on physical examination. The chest X-ray demonstrated left sided pleural effusion. Upon insertion of chest tube, 160 ml of transudative fluid was drained. CT performed detected intra-thoracic ELS in the lower postero-lateral part of the left lung (Fig. 1). Doppler ultrasonography revealed that blood supply to the sequestration was from the infra-diaphragmatic aorta. On postnatal 11th day, the sequestration was removed by thoracoscopic approach. Pathological examination also confirmed the diagnosis of pulmonary sequestration. There was no drainage from chest tube after the 3rd postoperative day. The chest tube was removed on 5th postoperative day and the patient was discharged. Currently, he is 8 months old and is normal in terms of growth and neuro-development.

**Figure:**
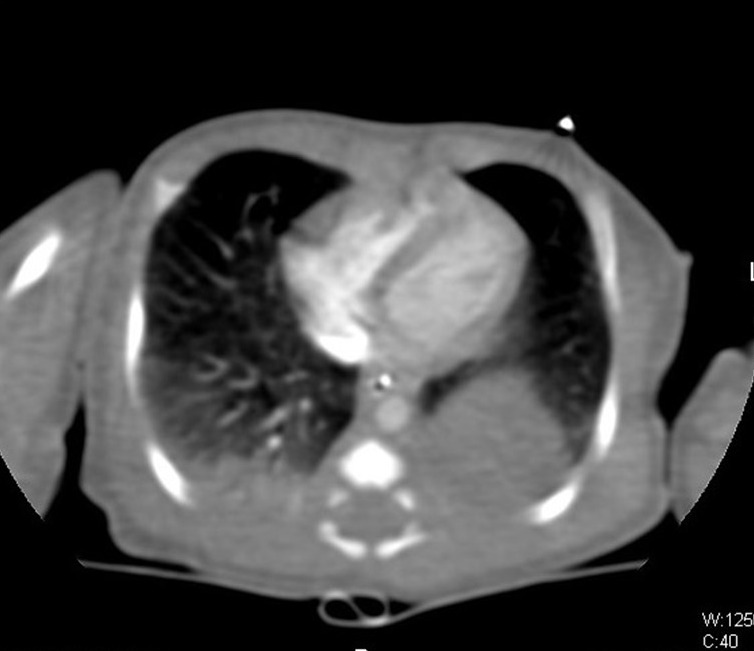
Figure 1:Chest CT showed intra-thoracic mass (extra-lobar sequestration) in the lower postero-lateral part of the left lung.

## DISCUSSION

Pulmonary sequestration is an uncommon congenital malformation of the lung that can be intra-lobar (sequestered part of lung lies within normal pulmonary visceral pleura) and extra-lobar (sequestration is completely separate and enclosed in its own pleural investment).[1] Approximately 90% of extra-lobar sequestrations occur in the left hemithorax and are generally asymptomatic during the neonatal period. The arterial blood supply to 80% ELS is through a direct branch of the thoracic or abdominal aorta, in 15% via another systemic artery and in 5% from the pulmonary artery.[2]

Primary fetal hydrothorax can resolve spontaneously in cases of primary, small, non-hydropic effusions. In more severe cases where hydramnios development is suspected, fetal therapy either thoracentesis or pleuro-amniotic shunting (PAS), is considered.[3,4] The lesion demonstrated in our patient was ELS which is generally asymptomatic in perinatal period, but, the patient required repeated thoracentesis in-utero due to fear of significant hydramnios and pleural effusion on serial US. In a recent review of 22 cases of ELS, prenatal treatment was needed in 12 cases, including PAS in 7 cases and thoracentesis in five.[5] In our case, antenatal diagnosis was not formed and the condition was diagnosed postnatally on CT scan. A fetal MRI would have made the diagnosis.

Thoracoscopic resection of pulmonary sequestration is believed to be the safest and effective method of treatment in an effort to minimize thoracotomies, postoperative pain, and scar. Apart from these disadvantages of thoracotomy, other complications such as winged scapula, problems of thoracic musculoskeletal development, vertebral scoliosis etc., can be avoided by thoracoscopic approach.[4] In conclusion, ELS may become symptomatic prenatally requiring fetal intervention to prevent hydrops fetalis. These lesions can be successfully managed thoracoscopically in neonatal life.

## Footnotes

**Source of Support:** Nil

**Conflict of Interest:** None declared

